# Development and Preliminary Assessment of a Tendon-Driven Thumb–Index Prosthesis with a Novel Hobbed-Pulley Actuation Mechanism

**DOI:** 10.3390/bioengineering13020197

**Published:** 2026-02-09

**Authors:** Patrícia Gomes, Pedro J. S. C. P. Sousa, João Nunes, Stephanos P. Zaoutsos, Susana Dias, Paulo J. Tavares, Pedro M. G. J. Moreira

**Affiliations:** 1 Instituto de Ciência e Inovação em Engenharia Mecânica e Engenharia Industrial (INEGI), Campus da FEUP, Rua Dr. Roberto Frias, 4200-465 Porto, Portugal; 2 Faculdade de Engenharia, Universidade do Porto, Rua Dr. Roberto Frias, 4200-465 Porto, Portugal; 3 Department of Energy Systems, University of Thessaly, 41500 Larisa, Greece

**Keywords:** thumb–index prosthesis, tendon-hobbed-pulley, current-based control

## Abstract

Prosthetic hands have seen significant improvements in recent years, enabling increasingly more natural interactions between patients with upper limb loss and their environment. Nonetheless, progress is continuously being made to enhance user acceptance, which remains a major drawback in such systems. The efficiency of the actuation mechanism is a critical parameter when designing these devices. Maximising actuation approach efficiency enables the use of smaller and lighter motors, thus decreasing the overall weight of the solution. Simultaneously, increased efficiency contributes to more precise motor control. Within this context, the present work introduces a novel actuation concept. Conventional tendon–pulley mechanisms are often susceptible to tendon slippage; therefore, a hobbed tendon–pulley approach was investigated to maintain cable tension more consistently and efficiently. This approach aims to provide smoother operation, improved reliability, and a reduced risk of mechanical failure due to tendon slippage. Simultaneously, the capability of holding and maintaining a set force is of utmost importance in these systems, and the force-feedback system is usually a major concern. The present work also focuses on comparing current and pressure-based control methodologies for the developed prosthesis. The current-based approach had the significant advantage of not requiring external sensors to be assembled on the prosthesis and not relying on the point of application of force being inside the sensor’s active area. During these tests, the prosthesis successfully grasped various objects of different sizes, shapes, stiffnesses, and weights using a current-based approach, without observable tendon slippage.

## 1. Introduction

The abandonment rate among prosthesis users is still far from ideal, despite the introduction of encouraging solutions in recent years. It is estimated that between 30% and 50% of prosthetic users do not use their devices regularly [1]. These numbers highlight the importance of ongoing, end-user-oriented innovation [2,3]. High abandonment rates are frequently attributed to several factors, including increased weight, higher costs, discomfort, difficulty of use and lack of sensory feedback [4].

Different actuation and user-centred approaches have been developed in recent years, trying to overcome these drawbacks. Linkage-driven and tendon–pulley mechanisms are the most commonly used actuation mechanisms. Soft approaches, differential mechanisms, and string actuators are also proposed in the literature.

Tendon–pulley approaches have been reported to offer low-cost manufacturing, low weight, and straightforward implementation of self-adaptive actuation [5]. Additionally, these systems can replicate smooth, lifelike finger articulation while ensuring efficient transmission of force [2,6].

This actuation approach is characterised by a tendon that passes through a series of attachment points in the finger structure [7,8,9,10]. Pulleys are frequently employed to pull and push the tendons. Some authors use a single tendon to perform extension and flexion movements, while approaches with increased complexity are also proposed.

To perform metacarpophalangeal (MCP), distal interphalanx (DIP), and proximal interphalanx (PIP) joints extension and flexion, a two-tendon–pulley system is proposed in [7]. The structure of this finger comprises extension springs and a friction clutch mechanism, which is used to alternate between static and dynamic friction states.

To create an underactuated 3 Degrees of Freedom (DoF) hand exoprosthesis, Estay et al. [11] employed a two-tendon transmission system mechanism. The thumb has two Degrees of Actuation (DoA), while the remaining DoA is responsible for the shared movement of the remaining fingers. Movements are performed sequentially since the same tendon is used to perform both extension/flexion and opposition/reposition movements. When one of the fingers is in contact with an object, it stops moving, and the remaining fingers continue to move due to the pulley system, resulting in an adaptive grasping [11].

A bidirectional passive transmission mechanism is proposed in [12]. Extension and flexion movements are performed by two different cables, attached to the extension and flexion wheels, respectively. The modification of the flexible ’fences’ allows for customising the dynamic response of each finger.

Laffranchi et al. [13] propose a 9 DoF solution by employing a tendon–pulley mechanism with two different groups of cables, with the PIP joint being locked in the design. The thumb is designed differently compared to the remaining fingers, as both IP and MCP joints are locked. Abduction movements are actuated while rotation is performed passively. Two tendons, a leader and a follower, routed inside the finger structure are proposed in this mechanism. Both the follower and leader tendons are under tension when the motor applies torque, which causes spring compression and torque generation in the MCP and PIP joints. The finger and the spring then stretch when the follower tendon slackens as the motor unwinds the leader tendon.

Highly actuated approaches are also proposed in the literature [8,14,15,16,17,18,19]. As an example, Yurova et al. [8] present a 21 DoF hand: four in each of the index, middle, ring, and little fingers and three in the thumb. Twenty-one servomotors are placed in the forearm, and threads are used to drive the phalanges that are interconnected by torsion springs.

Tian et al. [14] proposed a close biomimetic 3D-printed hand incorporating artificial human-like bones and ligaments. The entire finger structure was produced in elastic 3D printing resin. Biological ligament behaviour and muscle insertions were closely replicated. The motor directly actuates the MCP joint while the PIP and DIP joints are underactuated [14].

Gear sets are often combined with tendon–pulley approaches, allowing the motor to directly actuate the MCP joint, where the tendon drives the PIP and DIP joints. Jung et al. followed this approach and included an extension spring in the finger structure to allow for extension [20].

Another study, proposed by Liow et al., introduces a fully modular tendon-driven prosthetic hand, designed with interchangeable finger components that enable tool-free self-maintenance. The design incorporates a finger coupling and transmission system, which allows the fingers to be remotely actuated via DC motors housed within the palm and eliminates the need for tendon reconnection, making finger replacement easier and cost-effective [5].

Despite the aforementioned advantages, tendon–pulley actuation mechanisms are also associated with some drawbacks, with slippage being a commonly reported issue after prolonged operation. Energy losses resulting from friction are also frequently reported [21].

Based on this problem statement, the present work first aims to develop and validate a novel actuation mechanism that mitigates tendon slippage at tendon attachment points by integrating a geared transmission with a hobbed-pulley tendon drive. The second main goal is to implement and compare current-based and pressure-based control methodologies. Section 2 presents the mechanical development of the thumb–index prosthesis, including the novel actuation mechanism, while Section 3 focuses on the solution control. In Section 4, the main results and respective discussion are presented, while Section 5 and Section 6 outline the conclusions and future work, respectively.

## 2. Actuation Mechanism Design

### Mechanism Design

The design of the palm was prioritised early in the development process, as it served as the structural base for the prosthetic’s actuation system. It houses the motor, gear, and hobbed pulleys, which were treated as a single integrated unit due to their mechanical interdependence and the need for precise synchronisation. These three components form the core of the actuation mechanism.

Fused Deposition Modelling (FDM) was selected as the primary manufacturing method during the design stages for greater flexibility during the prototyping phase, enabling quick design modifications and iterations with minimal material waste. Polyethene Terephthalate Glycol (PETG) was used throughout the printing process due to its durability, good impact resistance, and low warping tendency, making it a suitable choice for functional prototypes that require both strength and dimensional stability [22].

A Faulhaber Series 1024K006SR brushed DC motor (Dr. Fritz Faulhaber GmbH & Co. K, Schönaich, Germany) was selected based on the thumb–index requirements for nominal voltage, efficiency, torque output, power consumption, and dynamic response. Several motors from Faulhaber and Maxon manufacturers were evaluated; however, the 1024K006SR achieved the highest efficiency (up to 83%), ensuring minimal energy losses and maximising mechanical output while still satisfying all other requirements. The motor incorporates a Faulhaber PA2-100 encoder (Dr. Fritz Faulhaber GmbH & Co. K, Schönaich, Germany) and 64:1 gear reduction to increase the torque output, reducing the rotational speed, and allowing the hand to generate sufficient force for multiple tasks, while enabling smooth, controlled, and precise motion necessary for fine motor control. Table 1 presents Faulhaber Series 1024K006SR brushed DC motor main specifications.

To avoid tendon slippage, hobbed pulleys were innovatively proposed in this design instead of the conventional pulleys commonly reported in the gear–cable hybrid mechanisms literature. Unlike standard pulleys, a hobbed pulley is a toothed component commonly employed in motion transmission systems, such as those found in 3D printers and filament feeders, and features a toothed section that is used to engage with the motor shaft and gear system. The hobbed pulley was used to transfer force through a cable-driven system, ensuring consistent cable tension and providing smooth, reliable actuation of the fingers. The grip provided by the toothed design enables a more secure transfer of force, which is crucial for replicating the natural movement and flexibility of the human hand with increased efficiency.

**Figure 1:**
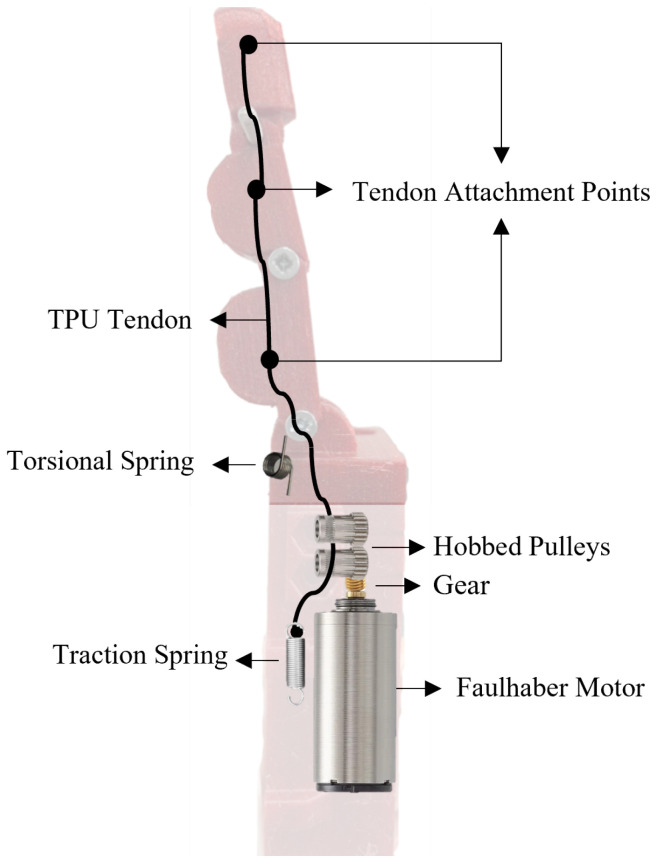
Schematic of the actuation mechanism and its main components proposed in this work.

Thermoplastic polyurethane (TPU), more specifically the commercial 3D printing filament Filaflex 82A (Recreus Industries S.L., Elda, Alicante, Spain) with a diameter of 1.75 mm [23], was selected as the tendon material due to its favourable mechanical, geometric properties and dimensional compatibility. The 1.75 mm filament matches the hobbed pulleys used in the design, improving engagement and helping to minimise wear and friction losses [24]. Although TPU is relatively stiffer compared to synthetic fibres like Dyneema or Kevlar, it allows for a smaller bending radius, which is particularly advantageous since tendons must be routed through tight and curved internal spaces [24,25]. TPU also offers excellent wear resistance and retains its mechanical integrity under repeated cycles of loading and bending, making it a suitable choice for long-term use. Its moderate stiffness provides structural stability without compromising flexibility, allowing for precise and controlled tendon actuation [24]. The tendon was fixed to the finger base with a traction spring whose range was carefully chosen to provide the necessary movement for full finger extension. The tendons are routed through the grooves of these pulleys ensuring optimal alignment and reducing slippage. As the pulley rotates, the tendon is either tensioned or released, thereby resulting in finger flexion or extension. To provide the fingers with a default resting position and ensure they return to that position after a flexion movement, a torsion spring was added between the phalanges. A schematic of the novel actuation mechanism proposed in the current work and its main components is presented in Figure 1.

**Figure 2:**
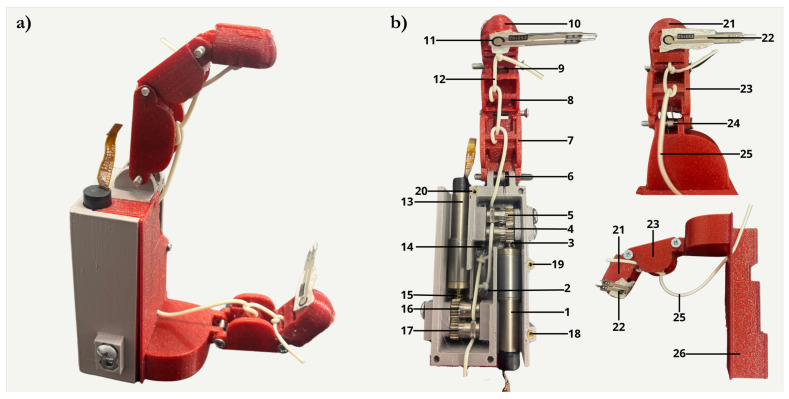
(**a**) Fully assembled prototype of the prosthetic hand, showing the integration of the thumb and index finger mechanisms in a compact configuration. (**b**) Internal palm structure showing the integrated actuation mechanism: Index finger: 1—Motor; 2—Traction spring; 3—Gear; 4, 5—Hobbed pulleys; 6, 9—Torsion spring; 7—Proximal phalanx; 8—Intermediate phalanx; 10—Distal phalanx; 11—Sensor; 12—Tendon. Thumb: 13—Motor; 14—Traction spring; 15—Gear; 16, 17—Hobbed pulleys; 21—Distal phalanx; 22—Sensor; 23—Proximal phalanx; 24—Torsion spring; 25—Tendon. 26—Cover for the palm and motor mounting; 18, 19 and 20—threaded inserts.

Figure 2a, shows the final configuration integrating all the mechanical components into a compact and cohesive structure. The thumb–index prototype offers a clear visual representation of the functional concept developed throughout this work. The structure of the index finger was based on the three anatomical phalanges found in the human hand: proximal, intermediate, and distal, while two phalanges were designed in the thumb: proximal and distal. Each phalanx was individually modelled to replicate the finger’s dimensions and kinematics while accommodating the mechanical constraints of the prosthetic system. Figure 2b illustrates the palm and fingers configuration as well as all the components and external sensors that enabled the experimental tests described in the following sections, in which the system’s performance and behaviour were assessed.

## 3. System Control

**Figure 3:**
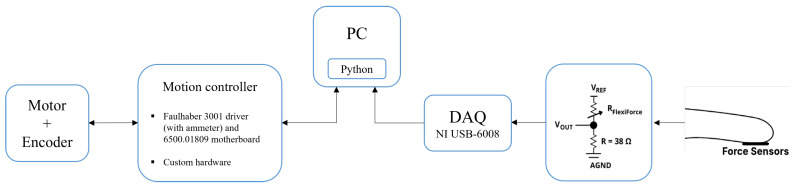
Overview of the control system architecture.

The diagram in Figure 3 presents an overview of the architecture of the developed prototype, which was designed to integrate actuation and sensory feedback, with each motor being independently controlled.

Two different approaches were used to implement the motor current-based control approach. The first employed a Faulhaber MC 3001 controller (Dr. Fritz Faulhaber GmbH & Co. K, Schönaich, Germany) with the 6500.01809 motherboard, while the second relied on a custom-developed electronic motor drive system. This step was essential to validate and optimise the custom-developed electronics for the NerveRepack project. A Python script (version 3.10), was developed using the Faulhaber Moman library [26,27] to communicate with the Faulhaber motherboard, while the Arduino IDE was used to control the motors using the custom-developed printed circuit board.

The Tekscan ESS102 (Tekscan, Inc., Norwood, MA, USA) [28] force sensor was used to apply the force sensor-based control approach. This sensors’ model, detailed in Table 2, was chosen due to its compact, circular shape with a diameter of 7.62 mm, making it ideal for integration into the prosthetic’s fingertips. Tekscan’s smaller sensors were excluded to prevent inaccurate measurements resulting from insufficient contact with objects. Force sensors were placed on the tips of the thumb and index finger, as presented before in Figure 2, allowing the system to detect contact with objects. A voltage-divider circuit recommended by the sensor manufacturer was implemented to acquire sensor data. The output of this circuit is connected to a National Instruments USB-6008 DAQ (National Instruments, Austin, TX, USA), acquiring at a rate of 500 Hz. Before validating the prototype, the sensors’ calibration curve was determined experimentally as

(1) V=0.214L+0.3681,

where *V* is the sensor output voltage (V) and *L* is the applied load (N); $R^{2}=0.9999$.

In both the current and force-based control approaches, each finger was controlled independently using a Proportional–Integral–Derivative (PID) controller operating at 10 Hz. The PWM (Pulsed-Width Modulation) output u(t) generated by the PID controller was defined by the following equation:

(2) $$u\left(t\right)=K_{p}\cdot e\left(t\right)+K_{i}\cdot \int e\left(\tau \right)\;d\tau +K_{d}\cdot \frac{de(t)}{dt},$$

where e(t) represents the error at the time, and $K_{p}$, $K_{i}$, and $K_{d}$ are the proportional, integral, and derivative gains, respectively. The PID controller for both fingers was configured with the following parameters: $K_{p}$ = 1000, $K_{i}$ = 20 and $K_{d}$ = 10 and the setpoint was adjusted during the testing phase for both index and thumb finger according to the object being handled. The high proportional gain was chosen to ensure a rapid system response and reduce the steady-state error. The integral gain was set to a moderate value to eliminate residual offset without causing excessive overshoot or instability, and the derivative gain was selected to attenuate oscillations and improve system stability by counteracting rapid changes in the error signal. In addition, a moving-average filter was applied to the measured signals. Once the target setpoint, current or force was reached, the motor maintained the grip for a few seconds before reversing direction to open the hand.

## 4. Results and Discussion

The prototype design and the current-based control approaches were evaluated. With respect to dimensions, the design was benchmarked against anthropomorphic hand data reported in the literature. In turn, current-based PID control algorithms were validated by assessing their ability to stabilise either the ammeter readings or the Tekscan sensor output, depending on the selected PID feedback signal. During preliminary testing, the hobbed pulleys were evaluated for their ability to prevent tendon slippage while avoiding observable damage to the tendon.

### 4.1. Prototype Dimensions

Prototype dimensions were extrapolated for five fingers and compared to the dimensions reported in the literature. The measured prototype dimensions are summarised in Table 3.

The prototype hand length (from the base of the palm to the tip of the index finger) was estimated to be approximately 164 mm. According to anthropometric data available in the literature, the average hand length is reported to vary between 180 and 198 mm, from the wrist to the tip of the middle finger [29]. Considering this, the measured length of the prototype falls within an acceptable range and can be regarded as a representative approximation of a medium-sized human hand.

Furthermore, the prototype has a width of 42 mm between the thumb and index finger. Assuming that the actuation mechanism of the five fingers occupies approximately 21 mm of width, the total estimated width across the palm would be around 105 mm. This exceeds the average palm width values reported in the literature, which range between 75 mm and 90 mm [29].

Moreover, the total prototype extrapolated mass was 385 g, which is adequate when compared with the values reported in the literature [30], indicating a promising balance between functionality and overall weight.

### 4.2. Control Approaches Validation

**Figure 4:**
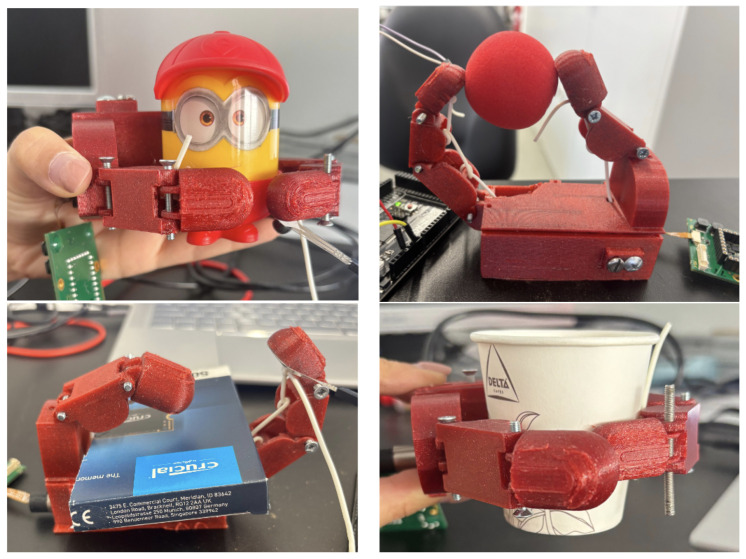
Hand grasping the objects used in the tests: figurine, foam ball, box, and paper cup.

Experimental tests were conducted by moving both index and thumb fingers. The procedure was carried out grasping the set of objects illustrated in Figure 4 with different stiffness, shape, and material properties: the foam ball was selected for its softness, the box for its geometric shape, the cup for its compliant material, and the figurine for its rigid body. The corresponding results obtained through these methodologies are presented in the subsequent sections.

#### 4.2.1. Current-Based Control

**Figure 5:**
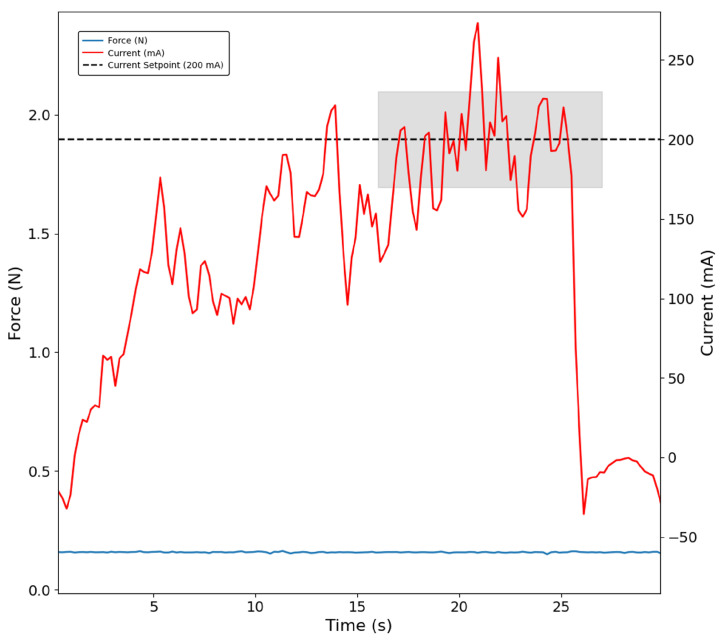
Index finger grasping the figurine with the current-based control approach. The shaded region indicates the interval during which the PID controller stabilises the motor current at 200 mA. No force signal is detected by the sensor (blue trace).

The current-based control approach was successfully validated, for example during the grasping of the figurine. It can be seen in Figure 5 that the current oscillates around the defined setpoint of 200 mA, as indicated in the shaded area. In the same figure, it is possible to observe that the sensor did not register any force, as the grasping position did not allow for the sensor to contact the object.

**Figure 6:**
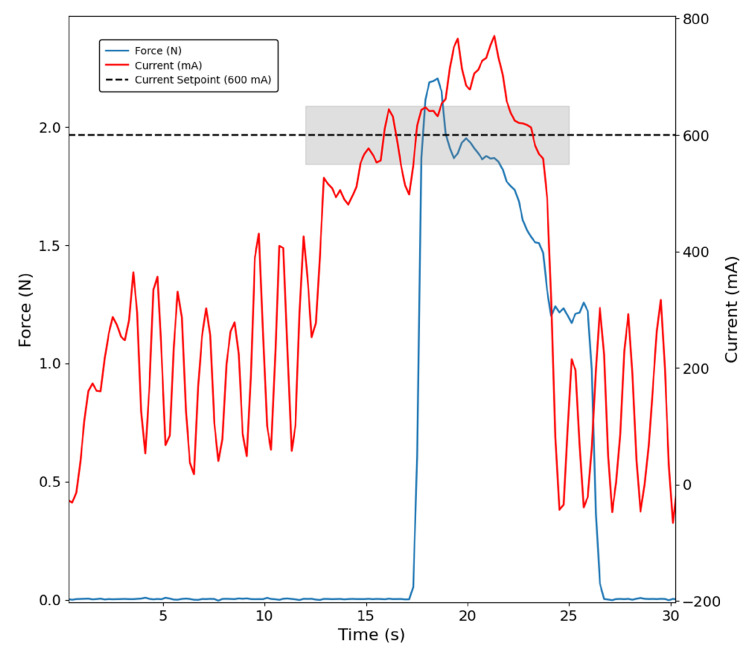
Thumb grasping the figurine with the current-based control approach. The shaded region indicates the interval during which the PID controller stabilises the motor current at 600 mA. Contact with the object is detected by the sensor (blue trace).

On the other hand, in Figure 6, the same control approach applied to the thumb while grasping the figurine resulted in increased noise; however, it remained approximately constant at the defined setpoint of 600 mA in the shaded region. Additionally, it can be seen that the peak current corresponds to the moment the sensor touches the object, followed by a drop in both current and force as the object is released. Before contact, motor current oscillates, likely due to suboptimal PID parameters or structural looseness in the prototype. This occurs because the measured current reflects all variations in friction and other losses associated with the kinematic chain. In turn, the thumb sensor has its spike only after some motor current has already been drawn.

**Figure 7:**
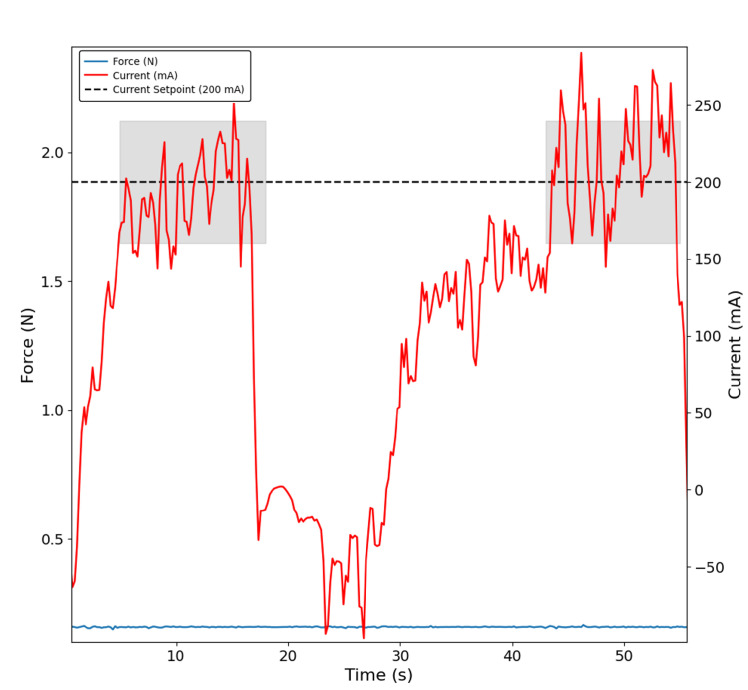
Index finger grasping the figurine in two distinct moments with the current-based control approach. The shaded region indicates the interval during which the PID controller stabilises the motor current at 200 mA. No force signal is detected by the sensor (blue trace).

**Figure 8:**
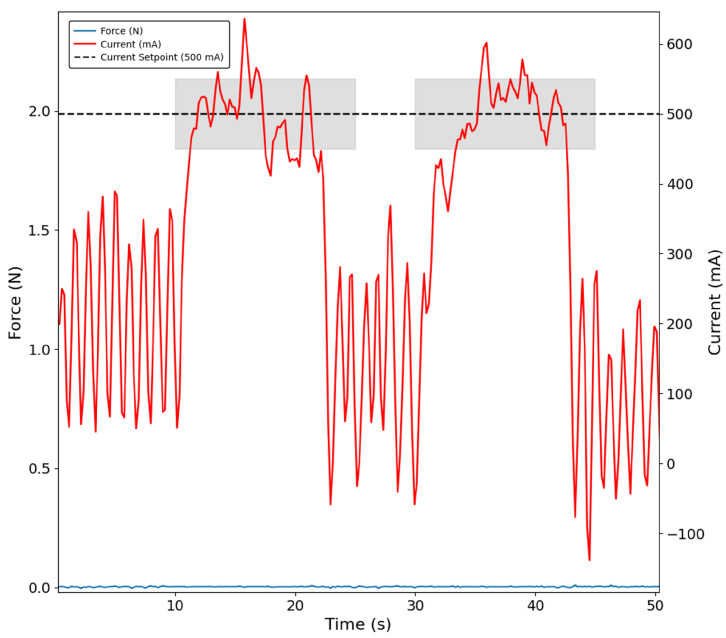
Thumb grasping the figurine in two distinct moments with the current-based control approach. The shaded region indicates the interval during which the PID controller stabilises the motor current at 500 mA. No force signal is detected by the sensor (blue trace).

The tests conducted with the remaining objects were very consistent with the results for the figurine case, highlighting both the inaccuracy of the index finger sensor in detecting contact with the object and the delayed detection in the thumb finger sensor when compared with the index finger. Additionally, some sequences of grasping were successfully implemented with a current-based control approach, as shown in Figure 7 and Figure 8, which confirms two distinct moments when, in this case, the paper box is grasped, and the control is capable of maintaining the defined current setpoint as highlighted in the shaded areas. These findings were consistently replicated across all remaining objects used in the testing protocol.

#### 4.2.2. Sensor-Based Control

**Figure 9:**
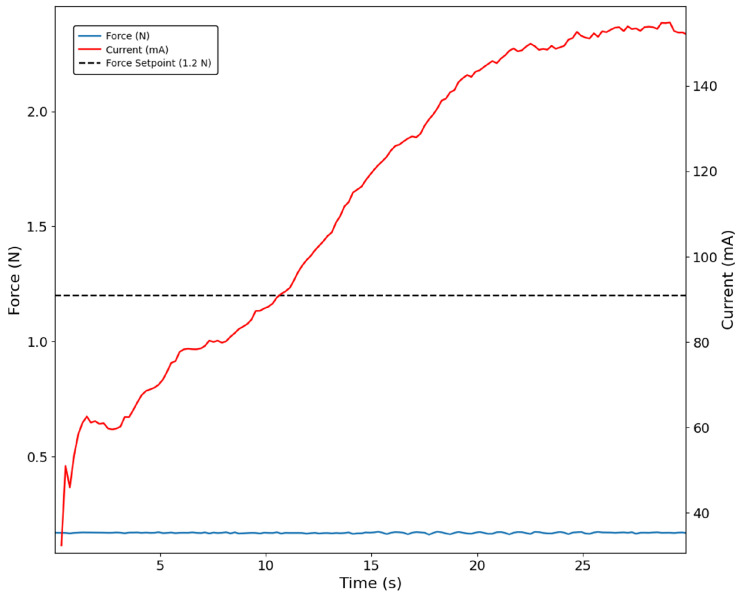
Index finger grasping the figurine with the force-based control approach. No force signal is detected by the sensor (blue trace). The motor current continuously increased (red trace).

**Figure 10:**
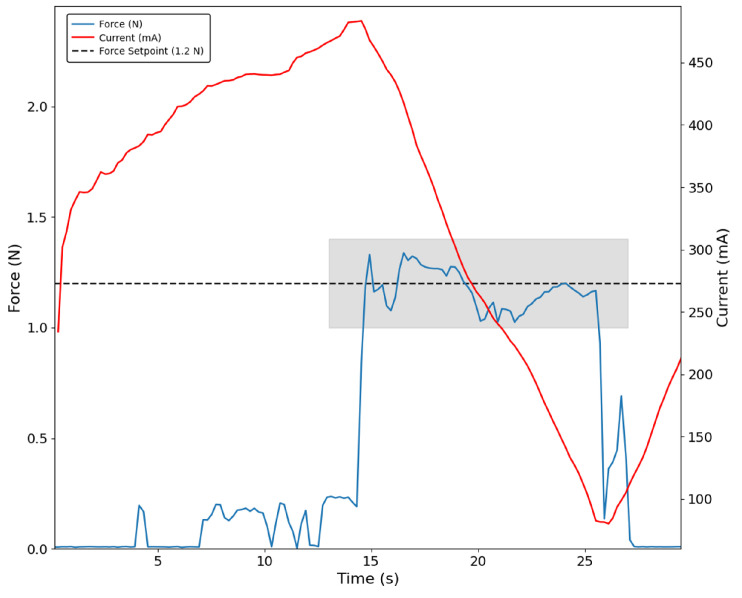
Thumb grasping the figurine with the force-based control approach. The shaded region indicates the interval during which the PID controller stabilises the force sensor output.

When grasping the figurine using a sensor-based approach in Figure 9, it is possible to confirm that the index force sensor never contacts the object, as the sensor signal remains approximately zero. For this reason, the sensor-based control was not effective for the index finger, and the current value continued to increase. Figure 10 shows that current begins to decrease when the sensor reaches its setpoint. When looking at the thumb data, it was once again confirmed that contact with the object is felt first in the motor than in the sensor, as confirmed by the delayed increase in sensor voltage compared to the motor current increase.

**Figure 11:**
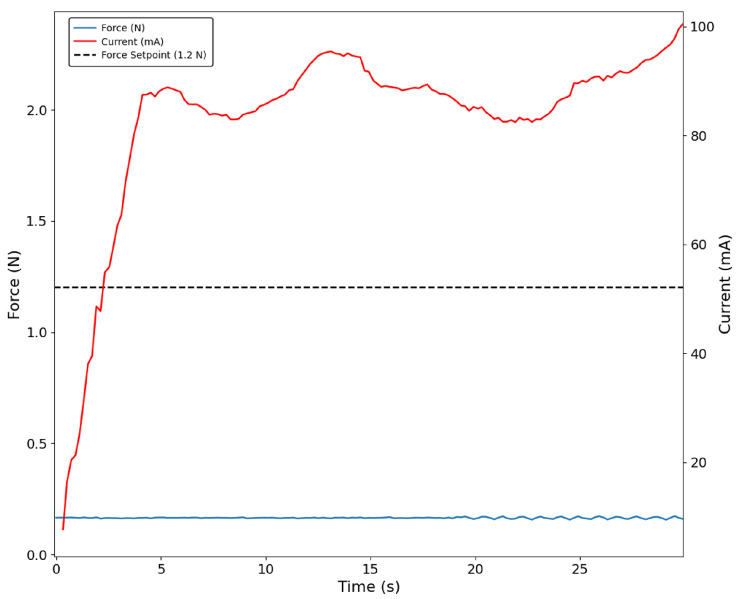
Index finger grasping the paper box with the force-based control approach. No force signal is detected by the sensor (blue trace).

The same behaviour was found in the tests developed with the paper cup. Once again, the fingertip sensor did not make contact with the object, and therefore, no force was recorded. Since the cup deforms while the figurine does not, it is also possible to obtain a constant motor current for a more extended period of time, as shown in Figure 11.

**Figure 12:**
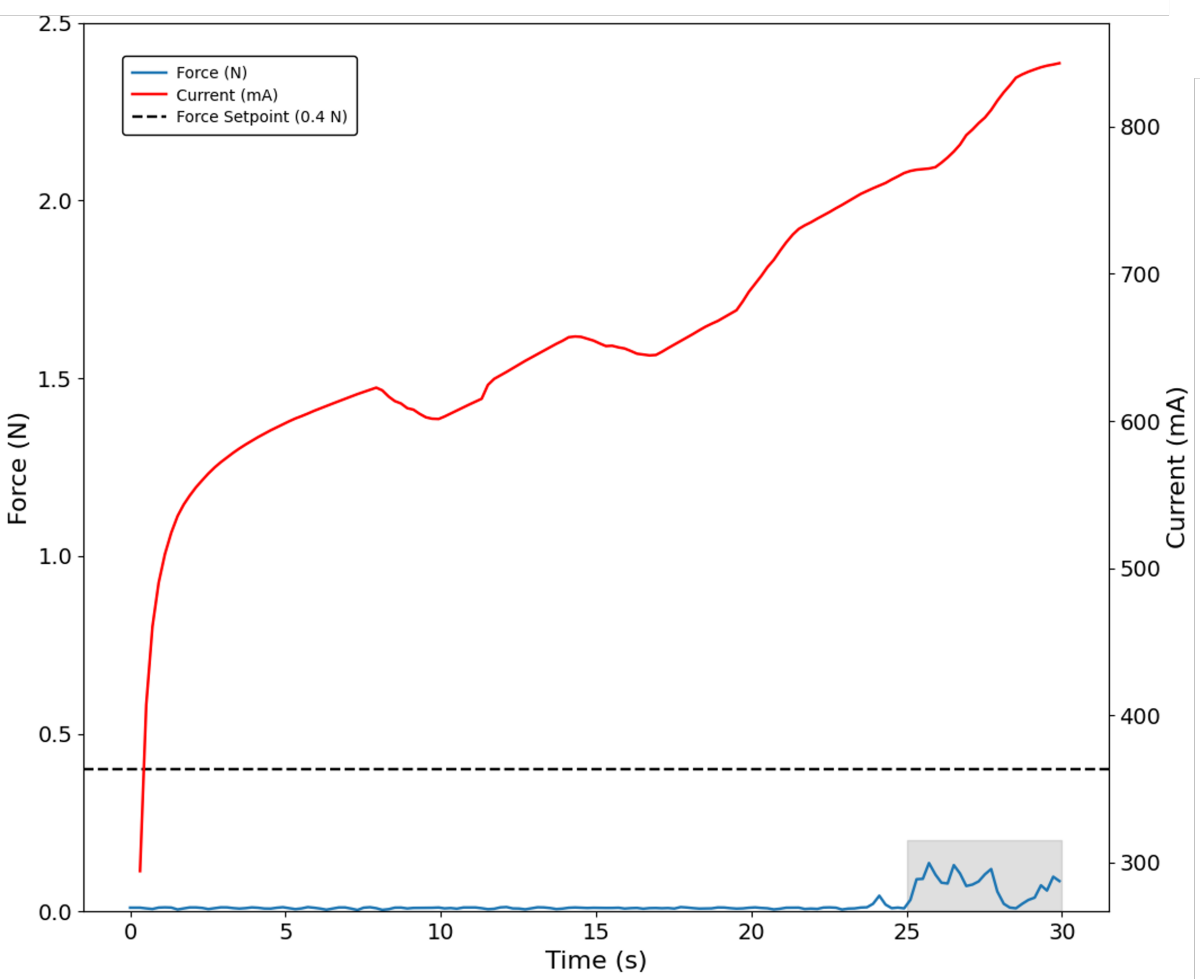
Thumb grasping the foam ball with the force-based control approach. Late contact is detected by the sensor at higher loads (shaded region).

Finally, when grasping the foam ball, which was the softest object, the thumb sensor was only capable of detecting the ball at higher loads, as demonstrated by the blue curve in the shaded area. In turn, motor current increased during the whole grasping procedure (Figure 12).

## 5. Conclusions

A functional thumb–index prosthetic prototype was successfully developed and implemented using a novel actuation mechanism that, to the best of the author’s knowledge, has not been previously reported in the literature. The proposed approach combines gears with hobbed pulleys to guide the tendon and transmit actuation forces, enabling the grasping of various objects without observable tendon slippage and damage during experimental trials.

The prototype’s size was compared to the average dimensions of a human hand. Its overall length was found to be slightly below the average but still within an acceptable range. However, the palm width has an estimated palm size of approximately 105 mm, exceeding typical dimensions reported in the literature. Moreover, the final prototype weighs an estimated total of around 385 g, which is slightly lower than values reported in the literature.

To evaluate the functionality of the prototype, two control strategies were implemented and tested. The first approach relied on monitoring the motor current, using a PID controller to regulate the effort applied during object interaction. The second strategy used the output of a force sensor as feedback in the PID loop, enabling the system to adjust based on the detected pressure.

Based on the results, the current-based control strategy demonstrated more promising and consistent performance compared to the sensor-based approach. One key advantage is its independence from sensor placement and the material properties of the object, which makes it especially suitable for handling soft or deformable materials. In contrast, the sensor-based control method exhibited several limitations because its performance heavily depends on consistent physical contact between the sensor and the object, which was not always guaranteed due to grasp configurations, object geometries, and materials. During most of the tests, the sensor failed to make contact, rendering the control ineffective. These findings emphasise not only the robustness of current-based control but also the critical role of sensor placement in achieving a successful sensor-based control approach.

## 6. Future Work

Several improvements can be implemented in the presented prototype. Firstly, the final version should not only rely on 3D printing as the primary manufacturing method. While 3D printing is well-suited for rapid prototyping and design iteration, it revealed that it does not provide the mechanical strength, precision, or durability required for long-term use. The selection of compatible materials is crucial to reduce wear, improve mechanical performance, and ensuring smoother movement over prolonged usage. Additionally, individual tuning of the PID controller for each finger would help reduce noise and improve control accuracy. Finally, regarding sensor-based control, several improvements should be made to the sensor placement to enhance this control approach.

Additionally, a more comprehensive experimental campaign will be carried out to quantitatively benchmark the proposed hobbed-pulley tendon actuation mechanism against conventional tendon pulley mechanisms reported in the literature. This evaluation should include measurements of tendon slippage, transmission efficiency, and non-ideal behaviours such as backlash and hysteresis during repeated loading and unloading cycles. Long-term operation and durability will also be considered.

## Figures and Tables

**Table 1:** Faulhaber DC motor 1024K006SR specifications.

Motor	Stall Current (A)	Nominal Torque After Reduction Ratio (mNm)	No Load Speed After Reduction (rpm)	Nominal Speed After Reduction Ratio (rpm)	Efficiency Max (%)
Faulhaber DC 1024K006SR	1.09	1442.56	13.84	8.33	83.00

**Table 2:** Tekscan ESS102 sensor specifications.

Model	Supplier	Size	Force Range	Repeatability	Price (€)	Ref.
ESS102	Tekscan	7.62 mm	4.53 kg (44 N)	<2.5%	10	[28]

**Table 3:** Measured dimensions of the final prototype for thumb, index finger, and palm.

Finger	Region	Length (±0.005 mm)	Width (±0.005 mm)
—	Palm	80	42
Index	Proximal phalanx	36	15
	Intermediate phalanx	28	15
	Distal phalanx	20	12
Thumb	Proximal phalanx	33	17
	Distal phalanx	25	19

## Data Availability

The datasets presented in this article are not readily available due to time limitations. Requests to access the datasets should be directed to the corresponding author.

## Abbreviations

The following abbreviations are used in this manuscript:

MCP	Metacarpophalangeal
DIP	Distal Interphalanx
PIP	Proximal Interphalanx
DoF	Degrees of Freedom
DoA	Degrees of Actuation
FDM	Fused Deposition Modelling
PETG	Polyethene 105 Terephthalate Glycol
TPU	Thermoplastic polyurethane
PID	Proportional–Integral–Derivative
PWM	Pulsed–Width Modulation

## References

[1] Witteveen H.J., de Rond L., Rietman J.S., Veltink P.H. (2012). Hand-opening feedback for myoelectric forearm prostheses: Performance in virtual grasping tasks influenced by different levels of distraction. J. Rehabil. Res. Dev..

[2] Ubosi D. (2023). Robotic prosthetic hands—A review. TechRxiv.

[3] Biddiss E., Chau T. (2007). Upper limb prosthesis use and abandonment: A survey of the last 25 years. Prosthetics Orthot. Int..

[4] Espinosa M., Nathan-Roberts D. (2019). Understanding Prosthetic Abandonment. Proc. Hum. Factors Ergon. Soc..

[5] Liow L., Clark A.B., Rojas N. (2020). OLYMPIC: A modular, tendon-driven prosthetic hand with novel finger and wrist coupling mechanisms. IEEE Robot. Autom. Lett..

[6] Vertongen J., Kamper D.G., Smit G., Vallery H. (2021). Mechanical Aspects of Robot Hands, Active Hand Orthoses, and Prostheses: A Comparative Review. IEEE/ASME Trans. Mechatron..

[7] Yong X., Zhu S., Sun Z., Chen S., Togo S., Yokoi H., Jing X., Li G. (2023). Highly Anthropomorphic Finger Design With a Novel Friction Clutch for Achieving Human-Like Reach-and-Grasp Movements. IEEE Trans. Neural Syst. Rehabil. Eng..

[8] Yurova V.A., Velikoborets G., Vladyko A. (2022). Design and Implementation of an Anthropomorphic Robotic Arm Prosthesis. Technologies.

[9] Zhou H., Tawk C., Alici G. (2022). A 3D Printed Soft Robotic Hand With Embedded Soft Sensors for Direct Transition Between Hand Gestures and Improved Grasping Quality and Diversity. IEEE Trans. Neural Syst. Rehabil. Eng..

[10] Vergaray R.A., Del Aguila R.F., Avellaneda G.A., Palomares R., Cornejo J., Cornejo-Aguilar J.A. Mechatronic System Design and Development of iROD: EMG Controlled Bionic Prosthesis for Middle-Third Forearm Amputee. Proceedings of the 2021 IEEE Fifth Ecuador Technical Chapters Meeting (ETCM).

[11] Estay D., Basoalto A., Ardila J., Cerda M., Barraza R. (2021). Development and implementation of an anthropomorphic underactuated prosthesis with adaptive grip. Machines.

[12] Chen T., Zhao X., Ma G., Tao B., Yin Z. (2021). Design of 3D-printed Cable Driven Humanoid Hand Based on Bidirectional Elastomeric Passive Transmission. Chin. J. Mech. Eng..

[13] Laffranchi M., Boccardo N., Traverso S., Lombardi L., Canepa M., Lince A., Semprini M., Saglia J.A., Naceri A., Sacchetti R. (2020). The Hannes hand prosthesis replicates the key biological properties of the human hand. Sci. Robot..

[14] Tian L., Zheng J., Thalmann N.M., Li H., Wang Q., Tao J., Cai Y., Magnenat Thalmann N., Li H., Wang Q. (2021). Design of a Single-Material Complex Structure Anthropomorphic Robotic Hand. Micromachines.

[15] Dasanayake N.P., Viduranga P.K.P., Perera U.L.S., Siyambalagoda S.A.P.K., Cooray T.M.G.C.S.P., Fernando K.R.T., Ranaweera R.K.P.S., Gopura R.A.R.C. iGrasp Hand: A Biomimetic Transradial Robotic Hand Prosthesis with a Clutching Mechanism. Proceedings of the 2021 Moratuwa Engineering Research Conference (MERCon).

[16] Adejumo D.O., Fadare D.A., Kazeem R.A., Ikumapayi O.M., Falana A., Adedayo A.S., Fadare D.A., Adeoye A.O.M., Ogundipe A.T., Olarinde E.S. (2023). A Low-Cost, Modular, Cable-Driven, Anthropomorphic Robotic Hand: A Conceptual Design and Application in Biomimetic Study. J. Eur. Des Syst. Autom..

[17] García C., Reyes A., Canul M., Gurza O., Cruzado S.S., Díaz J., Brieva J., Moya-Albor E., Ponce H., Garcia C. SCOMA hand prosthetic. Proceedings of the 2021 International Conference on Mechatronics, Electronics and Automotive Engineering (ICMEAE).

[18] Fajardo J., Ferman V., Cardona D., Maldonado G., Lemus A., Rohmer E. (2020). Galileo hand: An anthropomorphic and affordable upper-limb prosthesis. IEEE Access.

[19] Wu C., Song T., Wu Z., Cao Q., Fei F., Yang D., Xu B., Song A. (2021). Development and Evaluation of an Adaptive Multi-DOF Finger with Mechanical-Sensor Integrated for Prosthetic Hand. Micromachines.

[20] Jung S.Y., Kim S.G., Kim J.H., Park S.H. (2021). Development of Multifunctional Myoelectric Hand Prosthesis System with Easy and Effective Mode Change Control Method Based on the Thumb Position and State. Appl. Sci..

[21] Kashef S.R., Amini S., Akbarzadeh A. (2020). Robotic hand: A review on linkage-driven finger mechanisms of prosthetic hands and evaluation of the performance criteria. Mech. Mach. Theory.

[22] Prajapati S., Sharma J.K., Kumar S., Pandey S., Pandey M.K. (2024). A review on comparison of physical and mechanical properties of PLA, ABS, TPU, and PETG manufactured engineering components by using fused deposition modelling. Mater. Today Proc..

[23] TPU Filament Filaflex 82A, Flexible Filament for 3D Printing. https://recreus.com/en/products/filaflex-82a?srsltid=AfmBOoqBE7jh-rhKO7imnCrGgV4MnO8U5edlTfBgSIB_mcBHJRGVQPOU.

[24] Zhou X., Spiers A.J. InstaGrasp: An Entirely 3D Printed Adaptive Gripper with TPU Soft Elements and Minimal Assembly Time. Proceedings of the 2023 IEEE/RSJ International Conference on Intelligent Robots and Systems (IROS).

[25] Horigome A., Endo G. (2016). Basic study for drive mechanism with synthetic fiber rope–investigation of strength reduction by bending and terminal fixation method. Adv. Robot..

[26] Faulhaber Group (2025). Faulhaber Pyton Moman Library.

[27] Die Faulhaber GmbH & Co. KG (2019). Tutorial on the MomanLib.

[28] Tekscan, Inc. (2025). FlexiForce ESS102 Sensor.

[29] Belter J.T., Dollar A.M. Performance Characteristics of Anthropomorphic Prosthetic Hands. Proceedings of the 2011 IEEE International Conference on Rehabilitation Robotics.

[30] Medina-Coello P., Salvador-Domínguez B., Badesa F.J., María J., Corral R., Plastrotmann H., Morgado-Estévez A. (2024). Anthropomorphic Robotic Hand Prosthesis Developed for Children. Biomimetics.

